# Supplementary material for: Eliminating separase inhibition reveals absence of robust cohesin protection in oocyte metaphase II
Source: EMBO J. 2025 Aug 5;44(18):5187–214. doi: 10.1038/s44318-025-00522-0 (PMC12436617; doi:10.1038/s44318-025-00522-0)
Supplement: Supplementary file 1 — Appendix [file 44318_2025_522_MOESM1_ESM.pdf]

Appendix for:

Eliminating separase inhibition reveals absence of robust cohesin protection in oocyte metaphase II

Safia El Jailani, Damien Cladière, Elvira Nikalayevich, Sandra A. Touati, Vera Chesnokova, Shlomo Melmed, Eulalie Buffin and Katja Wassmann

Table of contents

Appendix Figure S1..page 2

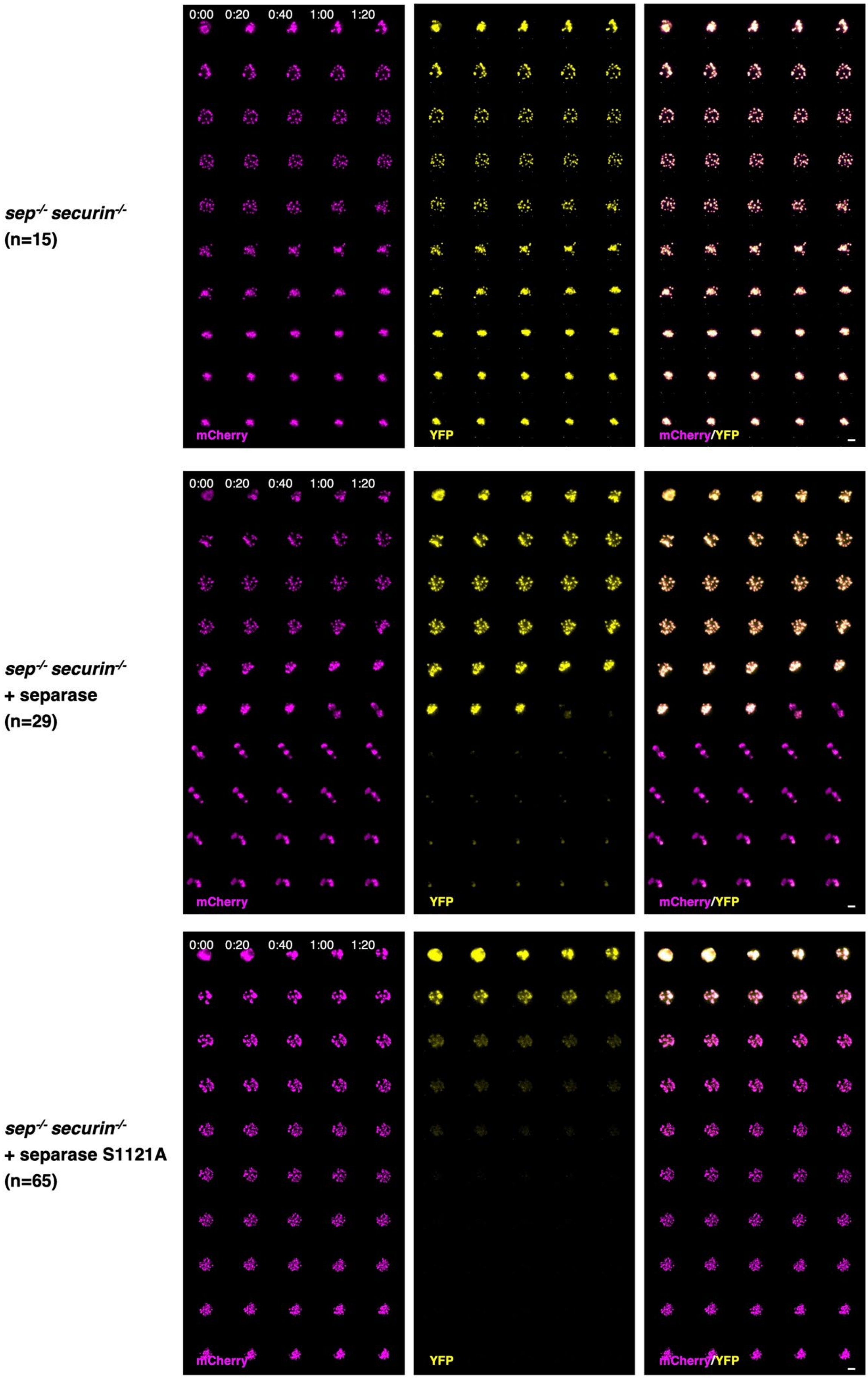

Appendix Figure S1 (related to Figure 3A and Figure EV3)

Complete loss of separase control reveals absence of cohesin protection in early prometaphase I

YFP and mCherry channels of selected time frames overlays shown in **Figure 3A**. Timepoints shown in hours (time after movie starts) and were taken every 20 mins, shown is the entire movie. n is the number of oocytes analysed. Scale bar (white) represents 20 µm.
