# Supplementary material for: Eliminating separase inhibition reveals absence of robust cohesin protection in oocyte metaphase II
Source: EMBO J. 2025 Aug 5;44(18):5187–214. doi: 10.1038/s44318-025-00522-0 (PMC12436617; doi:10.1038/s44318-025-00522-0)
Supplement: Supplementary file 3 — Movie EV1 [file 44318_2025_522_MOESM3_ESM.zip › Movie EV1/Legend Movie 1.docx]

S. El Jailani et al.

**Expanded View Movies - Figure legends**

**Movie EV1 (related to Figure 1B).**

Overlay of the YFP and mCherry channels of time lapse microscopy acquisitions of wild type (*sep^+/+^*) mouse oocytes expressing the cleavage sensor. Time after GVBD is shown in hours:minutes, and timepoints were taken every 20 mins, shown is the entire movie. Cleavage of the sensor is visible by the disappearance of the YFP signal from the chromosomes, whereas the mCherry signal remains localized to chromosomes. Scale bar (white) represents 20 μm.
