# Supplementary material for: Eliminating separase inhibition reveals absence of robust cohesin protection in oocyte metaphase II
Source: EMBO J. 2025 Aug 5;44(18):5187–214. doi: 10.1038/s44318-025-00522-0 (PMC12436617; doi:10.1038/s44318-025-00522-0)
Supplement: Supplementary file 4 — Movie EV2 [file 44318_2025_522_MOESM4_ESM.zip › Movie EV2/Legend Movie 2.docx]

S. El Jailani et al.

**Expanded View Movies - Figure legends**

**Movie EV2 (related to Figure 3A).**

Overlays of the YFP and mCherry channels of selected time frames shown in **Figure 3A**. *sep^−/−^ securin^−/−^* oocytes express the cleavage sensor and have been co-injected with mRNAs encoding for separase or separase S1121A, where indicated. Time after GVBD is shown in hours:minutes, and timepoints were taken every 20 mins, shown is the entire movie. Scale bar (white) represents 20 μm.
