# Supplementary material for: Eliminating separase inhibition reveals absence of robust cohesin protection in oocyte metaphase II
Source: EMBO J. 2025 Aug 5;44(18):5187–214. doi: 10.1038/s44318-025-00522-0 (PMC12436617; doi:10.1038/s44318-025-00522-0)
Supplement: Supplementary file 5 — Movie EV3 [file 44318_2025_522_MOESM5_ESM.zip › Movie EV3/Legend Movie 3.docx]

S. El Jailani et al.

**Expanded View Movies - Figure legends**

**Movie EV3 (related to Figure 5B).**

Time lapse acquisitions of the selected time frames shown in **Figure 5B** (bottom right). *sep^−/−^ securin^−/−^* oocytes were injected with separase S1121A around 16h after GVBD, and were subjected to live imaging around 18h after GVBD. Prior to acquisition, oocytes were preincubated in culture media containing SiR-DNA to visualize chromosomes. Time after start of the movie is shown in hours:minutes, shown is the entire movie. bar (white) represents 20 μm.
