# Supplementary figures and images for: Eliminating separase inhibition reveals absence of robust cohesin protection in oocyte metaphase II
Source: EMBO J. 2025 Aug 5;44(18):5187–214. doi: 10.1038/s44318-025-00522-0 (PMC12436617; doi:10.1038/s44318-025-00522-0)

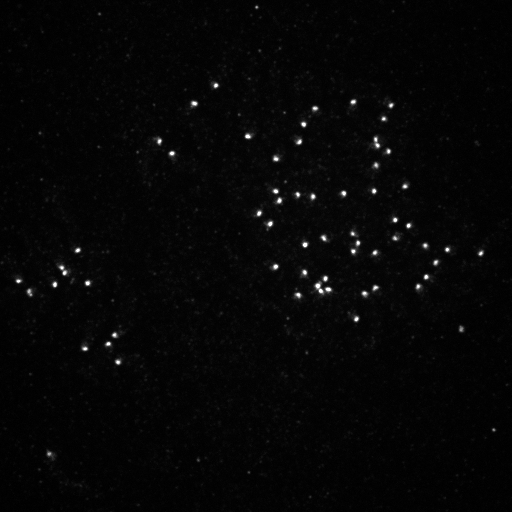

Supplement: Supplementary file 6 — Source data Fig. 1 [file 44318_2025_522_MOESM6_ESM.zip › Figure 1/1G/SepKO + sepS1121A Meta II.tif]

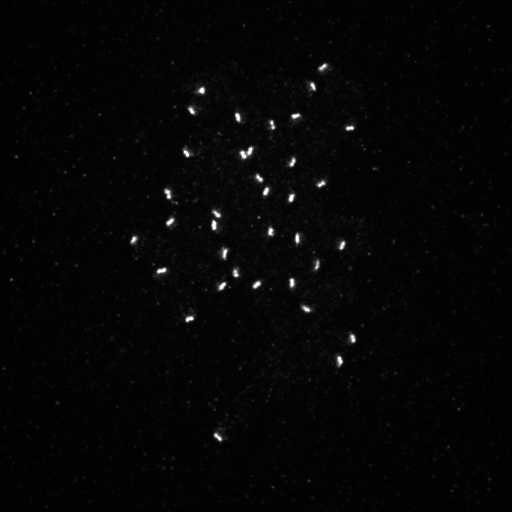

Supplement: Supplementary file 6 — Source data Fig. 1 [file 44318_2025_522_MOESM6_ESM.zip › Figure 1/1G/SepKO Meta II.tif]

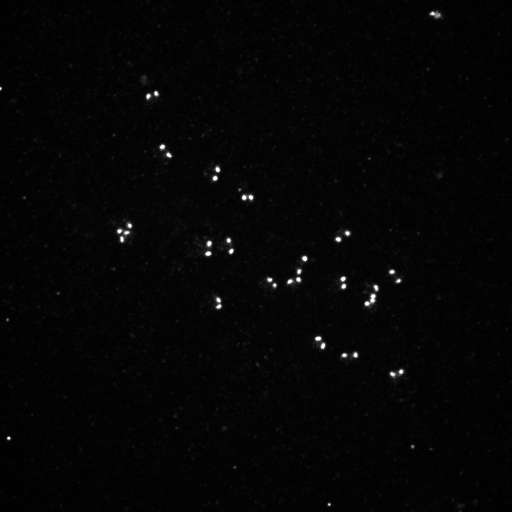

Supplement: Supplementary file 6 — Source data Fig. 1 [file 44318_2025_522_MOESM6_ESM.zip › Figure 1/1G/SepKO + sep wt Meta II.tif]

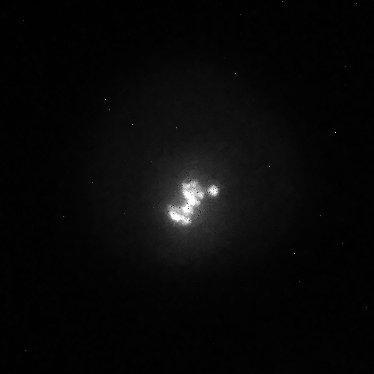

Supplement: Supplementary file 6 — Source data Fig. 1 [file 44318_2025_522_MOESM6_ESM.zip › Figure 1/1B/Sep+:+.tif]

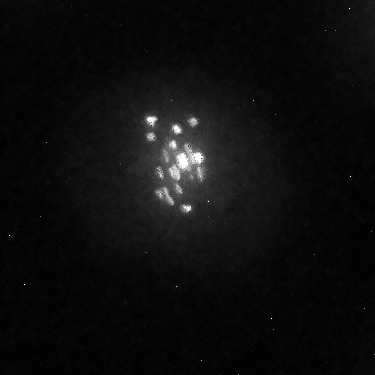

Supplement: Supplementary file 6 — Source data Fig. 1 [file 44318_2025_522_MOESM6_ESM.zip › Figure 1/1B/Sep+:+ + separase S1121A.tif]

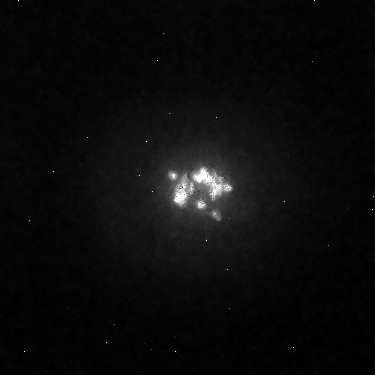

Supplement: Supplementary file 6 — Source data Fig. 1 [file 44318_2025_522_MOESM6_ESM.zip › Figure 1/1B/SepKO + separase S1121A.tif]

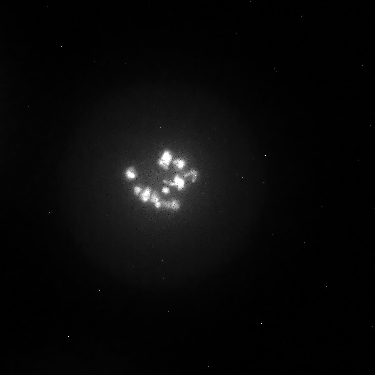

Supplement: Supplementary file 6 — Source data Fig. 1 [file 44318_2025_522_MOESM6_ESM.zip › Figure 1/1B/SepKO.tif]

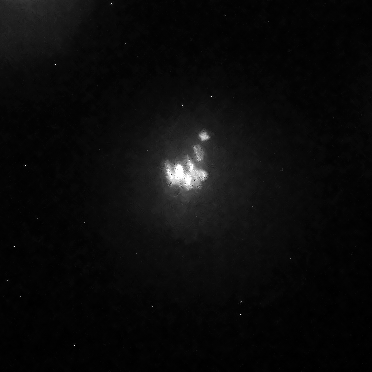

Supplement: Supplementary file 6 — Source data Fig. 1 [file 44318_2025_522_MOESM6_ESM.zip › Figure 1/1B/SepKO + wt separase.tif]

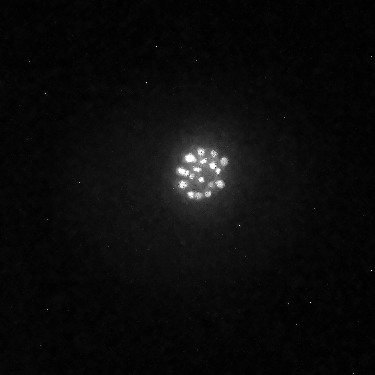

Supplement: Supplementary file 6 — Source data Fig. 1 [file 44318_2025_522_MOESM6_ESM.zip › Figure 1/1B/Sep+:+ + separase wt.tif]

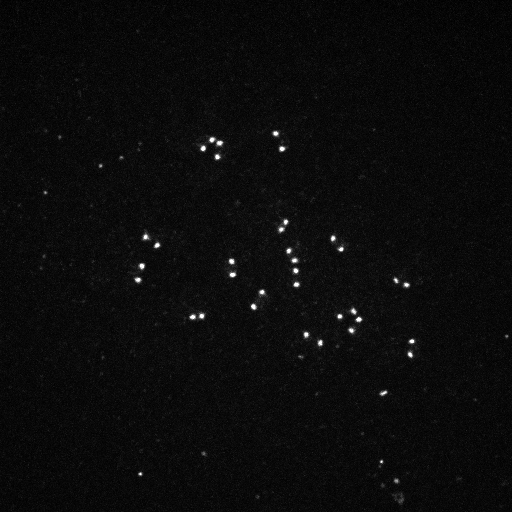

Supplement: Supplementary file 6 — Source data Fig. 1 [file 44318_2025_522_MOESM6_ESM.zip › Figure 1/1E/SepKO + wt sep MI Exit .tif]

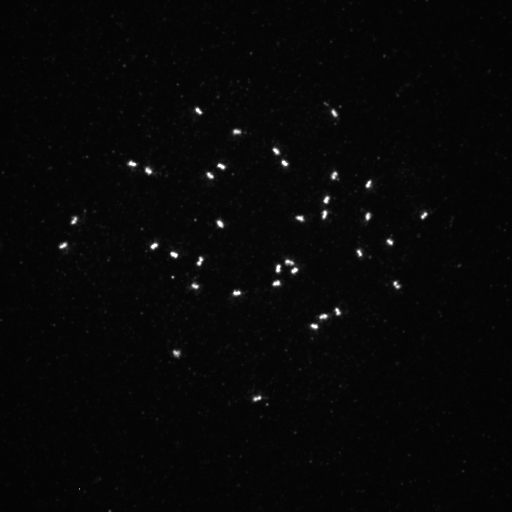

Supplement: Supplementary file 6 — Source data Fig. 1 [file 44318_2025_522_MOESM6_ESM.zip › Figure 1/1E/SepKO MI Exit.tif]

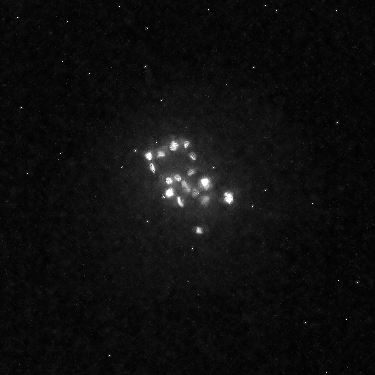

Supplement: Supplementary file 7 — Source data Fig. 2 [file 44318_2025_522_MOESM7_ESM.zip › Figure 2/2A/Securin+:+.tif]

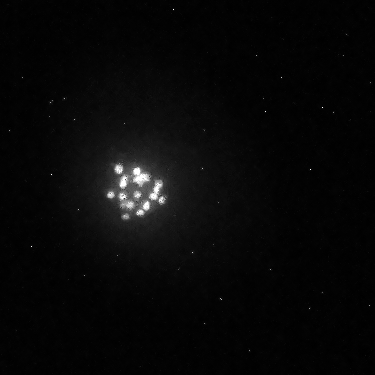

Supplement: Supplementary file 7 — Source data Fig. 2 [file 44318_2025_522_MOESM7_ESM.zip › Figure 2/2A/SecuKO.tif]

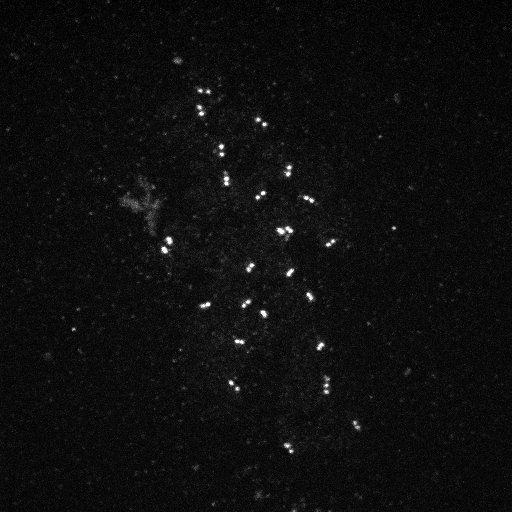

Supplement: Supplementary file 7 — Source data Fig. 2 [file 44318_2025_522_MOESM7_ESM.zip › Figure 2/2D/securin+:+ - MI Exit.tif]

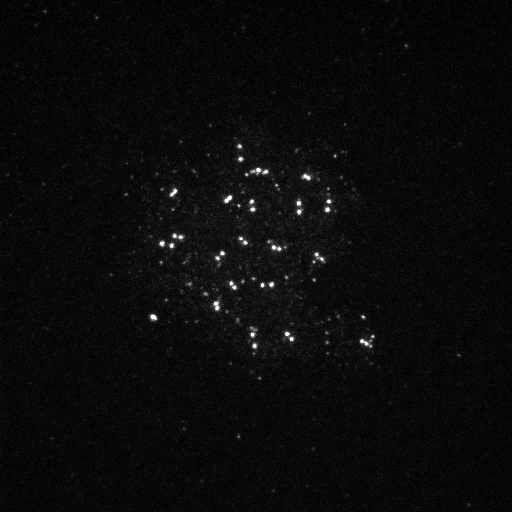

Supplement: Supplementary file 7 — Source data Fig. 2 [file 44318_2025_522_MOESM7_ESM.zip › Figure 2/2D/secuKO - MI Exit.tif]

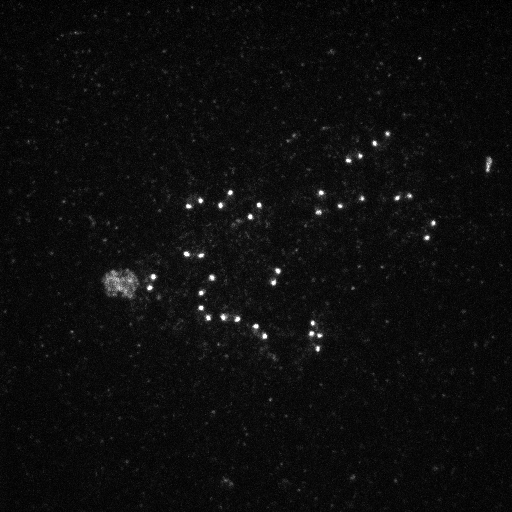

Supplement: Supplementary file 7 — Source data Fig. 2 [file 44318_2025_522_MOESM7_ESM.zip › Figure 2/2D/secuKO - Meta II.tif]

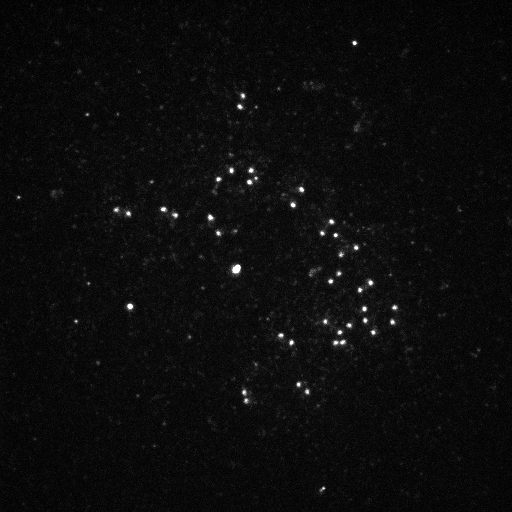

Supplement: Supplementary file 7 — Source data Fig. 2 [file 44318_2025_522_MOESM7_ESM.zip › Figure 2/2D/securin+:+ - Meta II.tif]

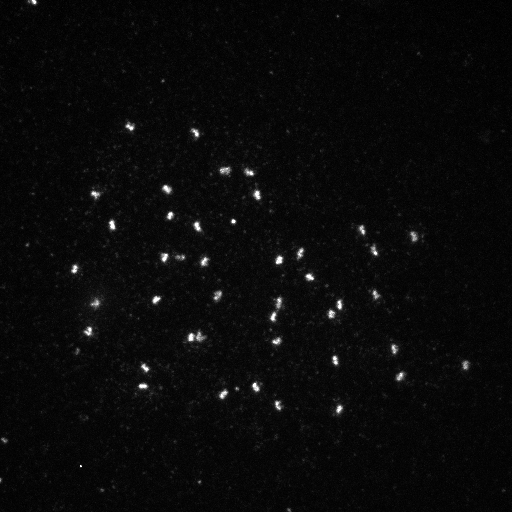

Supplement: Supplementary file 8 — Source data Fig. 3 [file 44318_2025_522_MOESM8_ESM.zip › Figure 3/3D/sep:secKO + sepS1121A + securin.tif]

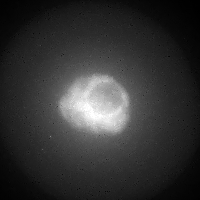

Supplement: Supplementary file 8 — Source data Fig. 3 [file 44318_2025_522_MOESM8_ESM.zip › Figure 3/3A/sep-:-securin-:- KO + separase S1121A.tif]

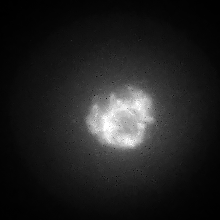

Supplement: Supplementary file 8 — Source data Fig. 3 [file 44318_2025_522_MOESM8_ESM.zip › Figure 3/3A/sep-:-securin-:- KO + wt separase.tif]

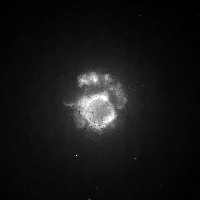

Supplement: Supplementary file 8 — Source data Fig. 3 [file 44318_2025_522_MOESM8_ESM.zip › Figure 3/3A/sep-:-securin-:- KO.tif]

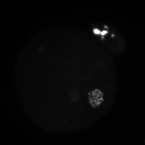

Supplement: Supplementary file 9 — Source data Fig. 4 [file 44318_2025_522_MOESM9_ESM.zip › Figure 4/4B/sep+:+ + separase S1121A.tif]

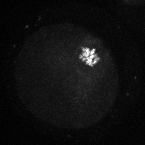

Supplement: Supplementary file 9 — Source data Fig. 4 [file 44318_2025_522_MOESM9_ESM.zip › Figure 4/4B/sepKO + separase S1121A.tif]

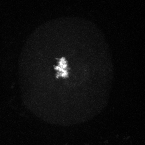

Supplement: Supplementary file 9 — Source data Fig. 4 [file 44318_2025_522_MOESM9_ESM.zip › Figure 4/4B/sepKO + wt separase.tif]

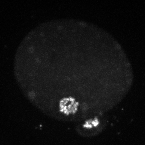

Supplement: Supplementary file 9 — Source data Fig. 4 [file 44318_2025_522_MOESM9_ESM.zip › Figure 4/4B/sep+:+ + wt separase.tif]

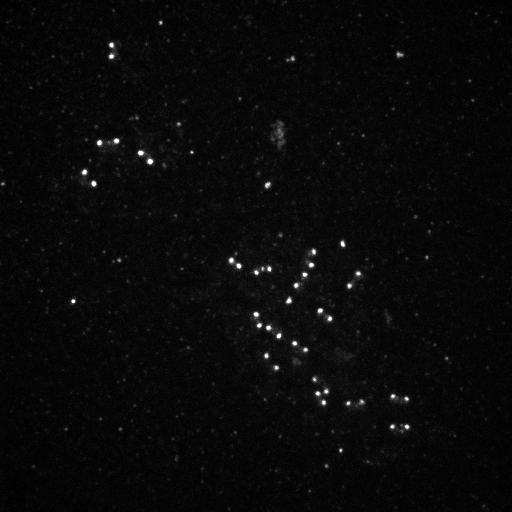

Supplement: Supplementary file 9 — Source data Fig. 4 [file 44318_2025_522_MOESM9_ESM.zip › Figure 4/4D/sep+:+ + sepS1121A.tif]

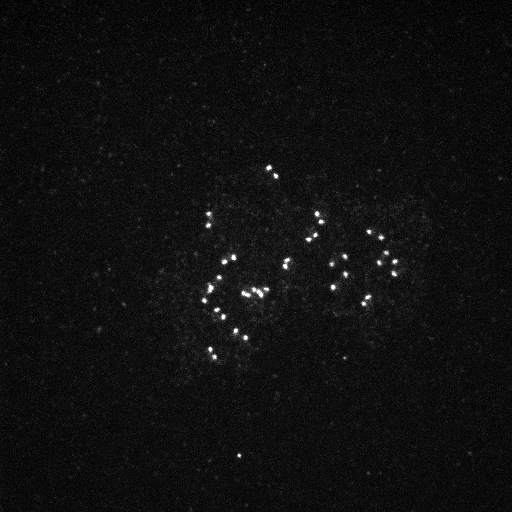

Supplement: Supplementary file 9 — Source data Fig. 4 [file 44318_2025_522_MOESM9_ESM.zip › Figure 4/4D/sep+:+.tif]

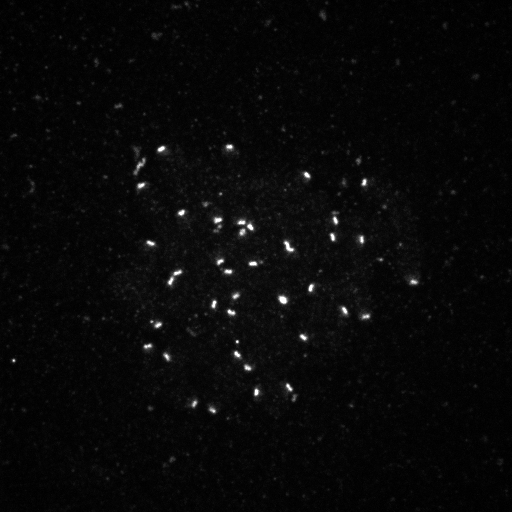

Supplement: Supplementary file 9 — Source data Fig. 4 [file 44318_2025_522_MOESM9_ESM.zip › Figure 4/4D/sepKO + sepS1121A.tif]

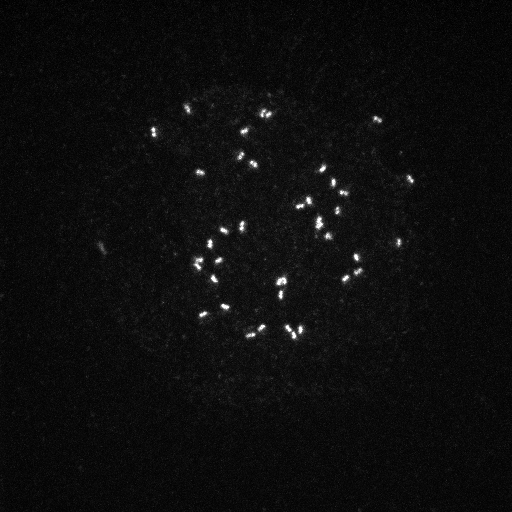

Supplement: Supplementary file 9 — Source data Fig. 4 [file 44318_2025_522_MOESM9_ESM.zip › Figure 4/4D/sepKO.tif]

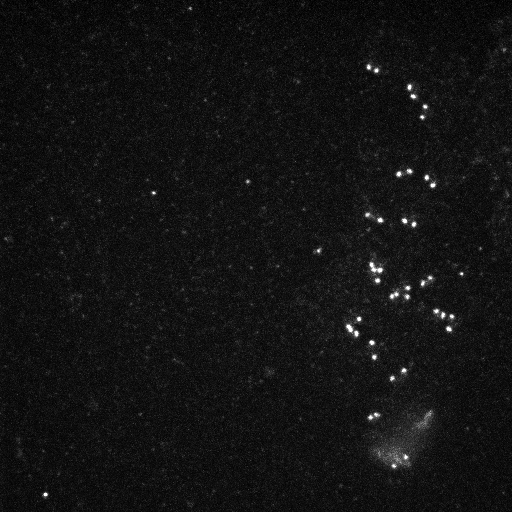

Supplement: Supplementary file 9 — Source data Fig. 4 [file 44318_2025_522_MOESM9_ESM.zip › Figure 4/4D/sep+:+ + sep wt.tif]

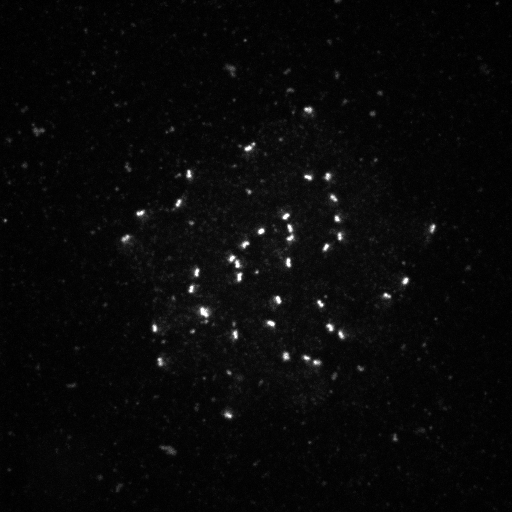

Supplement: Supplementary file 9 — Source data Fig. 4 [file 44318_2025_522_MOESM9_ESM.zip › Figure 4/4D/sepKO + sep wt.tif]

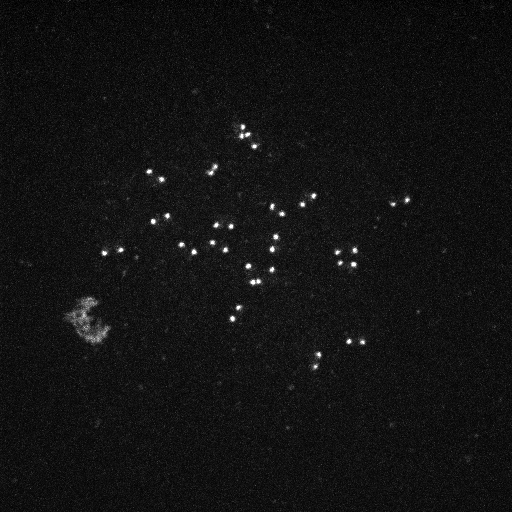

Supplement: Supplementary file 10 — Source data Fig. 5 [file 44318_2025_522_MOESM10_ESM.zip › Figure 5/5D/sep+:+ + sepS1121A.tif]

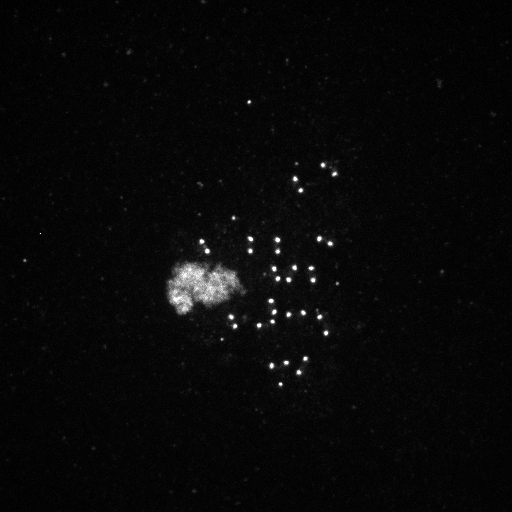

Supplement: Supplementary file 10 — Source data Fig. 5 [file 44318_2025_522_MOESM10_ESM.zip › Figure 5/5D/sep+:+.tif]

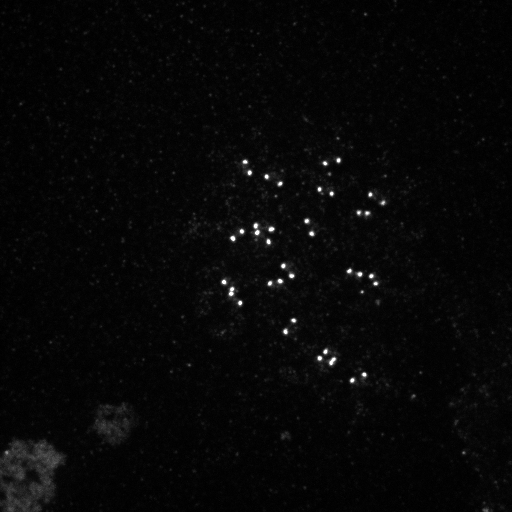

Supplement: Supplementary file 10 — Source data Fig. 5 [file 44318_2025_522_MOESM10_ESM.zip › Figure 5/5D/sep+:+ + sep wt.tif]

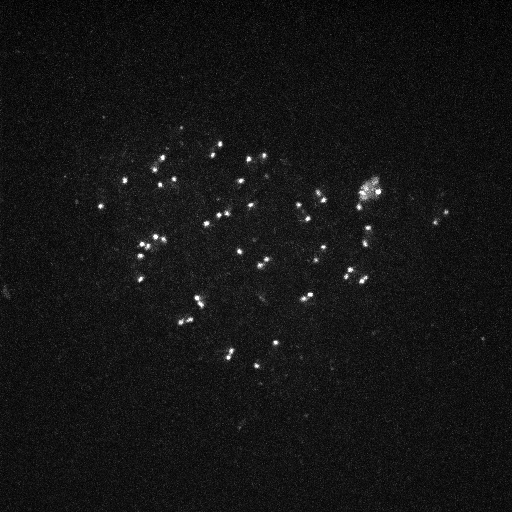

Supplement: Supplementary file 10 — Source data Fig. 5 [file 44318_2025_522_MOESM10_ESM.zip › Figure 5/5D/sep:secKO + sepS1121A.tif]

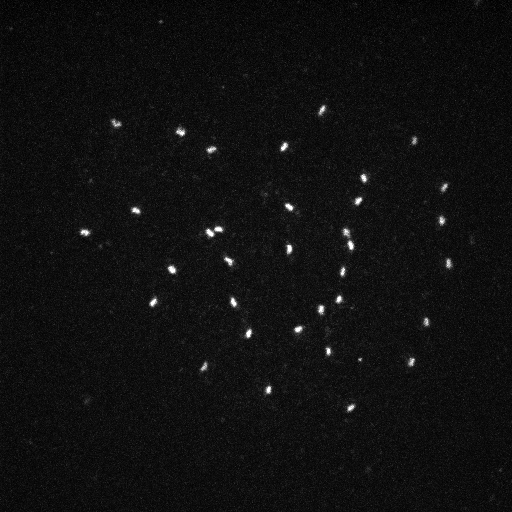

Supplement: Supplementary file 10 — Source data Fig. 5 [file 44318_2025_522_MOESM10_ESM.zip › Figure 5/5D/sep:secKO.tif]

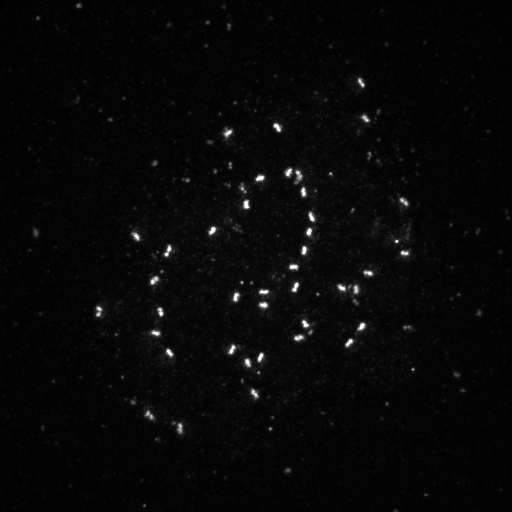

Supplement: Supplementary file 10 — Source data Fig. 5 [file 44318_2025_522_MOESM10_ESM.zip › Figure 5/5D/sep:secKO + sep wt.tif]

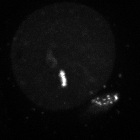

Supplement: Supplementary file 10 — Source data Fig. 5 [file 44318_2025_522_MOESM10_ESM.zip › Figure 5/5B/sep+:+ + separase S1121A.tif]

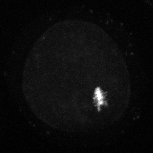

Supplement: Supplementary file 10 — Source data Fig. 5 [file 44318_2025_522_MOESM10_ESM.zip › Figure 5/5B/sep-:-securin-:- + wt separase.tif]

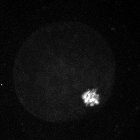

Supplement: Supplementary file 10 — Source data Fig. 5 [file 44318_2025_522_MOESM10_ESM.zip › Figure 5/5B/sep-:-securin-:- + separase S1121A.tif]

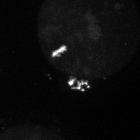

Supplement: Supplementary file 10 — Source data Fig. 5 [file 44318_2025_522_MOESM10_ESM.zip › Figure 5/5B/sep+:+ + wt separase.tif]

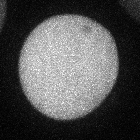

Supplement: Supplementary file 11 — Source data Fig. 6 [file 44318_2025_522_MOESM11_ESM.zip › Figure 6/6F/securinKO + separaseS1121A + STRONTIUM.tif]

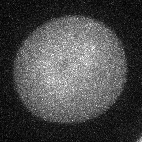

Supplement: Supplementary file 11 — Source data Fig. 6 [file 44318_2025_522_MOESM11_ESM.zip › Figure 6/6F/securinKO + separase S1121A.tif]

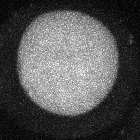

Supplement: Supplementary file 11 — Source data Fig. 6 [file 44318_2025_522_MOESM11_ESM.zip › Figure 6/6D/sep-:-securin-:- + separase S1121A.tif]

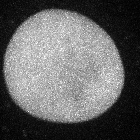

Supplement: Supplementary file 11 — Source data Fig. 6 [file 44318_2025_522_MOESM11_ESM.zip › Figure 6/6D/sep-:-securin-:- + separase S1121A + STRONTIUM.tif]
