# Supplementary material for: Eliminating separase inhibition reveals absence of robust cohesin protection in oocyte metaphase II
Source: EMBO J. 2025 Aug 5;44(18):5187–214. doi: 10.1038/s44318-025-00522-0 (PMC12436617; doi:10.1038/s44318-025-00522-0)
Supplement: Supplementary file 12 — Expanded View Figures [file 44318_2025_522_MOESM12_ESM.pdf]

# Expanded View Figures

**Figure EV1. Related to Fig. 1B.**

Cyclin B1-dependent inhibition of separase is not essential before meiosis I exit. (**A**) YFP and mCherry channels of the selected time frames overlays shown in Fig. 1B. Time after GVBD is shown in hours:minutes (anaphase I onset is indicated with an arrowhead). n is the number of oocytes analyzed. Scale bar (white) represents 20 µm. (**B**) Frequency of polar body extrusion (PBE) of *sep*$^{-/-}$ oocytes expressing separase or separase S1121A, shown in Fig. 1B. Total number of oocytes quantified for each condition, and of each category, is indicated. sep: separase. Data information: Results shown were obtained from at least three independent biological replicates.

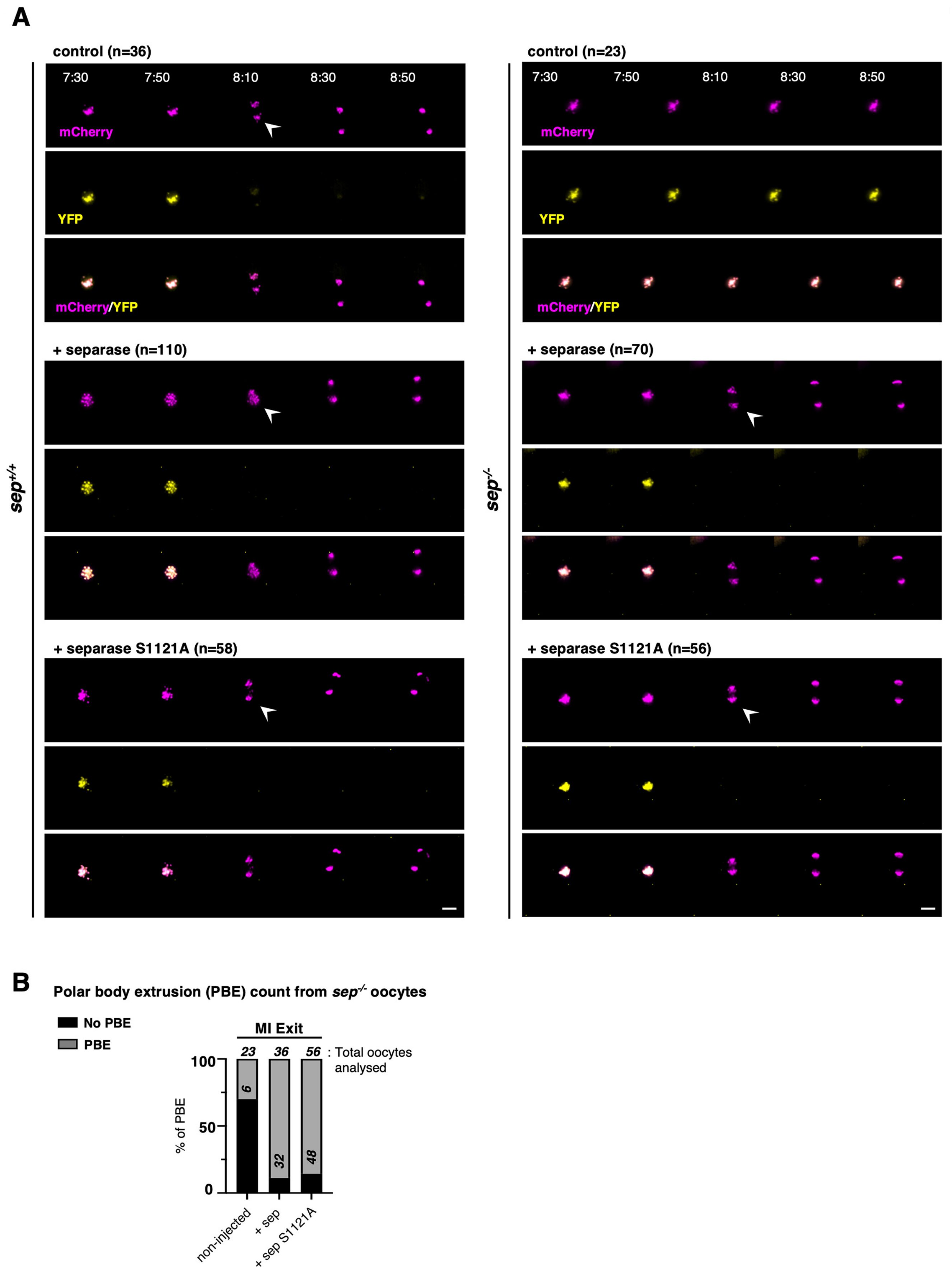
A
control (n=36)
7:30
7:50
8:10
8:30
8:50
mCherry
YFP
mCherry/YFP
+ separase (n=110)
+ separase S1121A (n=58)
sep+/+
control (n=23)
+ separase (n=70)
+ separase S1121A (n=56)
sep-/-
B
Polar body extrusion (PBE) count from sep-/- oocytes
No PBE
PBE
MI Exit
23
36
56
: Total oocytes analysed
100
50
0
% of PBE
6
32
48
non-injected
+ sep
+ sep S1121A

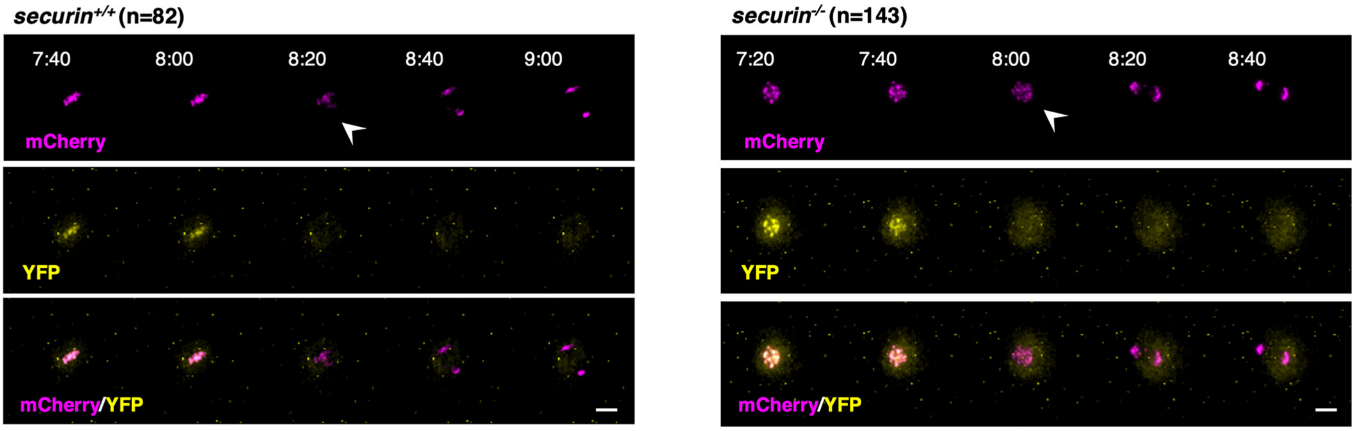


**Figure EV2. Related to Fig. 2A.**

Securin is not essential for separase inhibition in meiosis I. YFP and mCherry channels of the selected time frames overlays shown in Fig. 2A. Time after GVBD is shown in hours:minutes (anaphase I onset is indicated with an arrowhead). *n* is the number of oocytes analyzed. Scale bar (white) represents 20 µm. Data information: Results shown were obtained from at least three independent biological replicates.

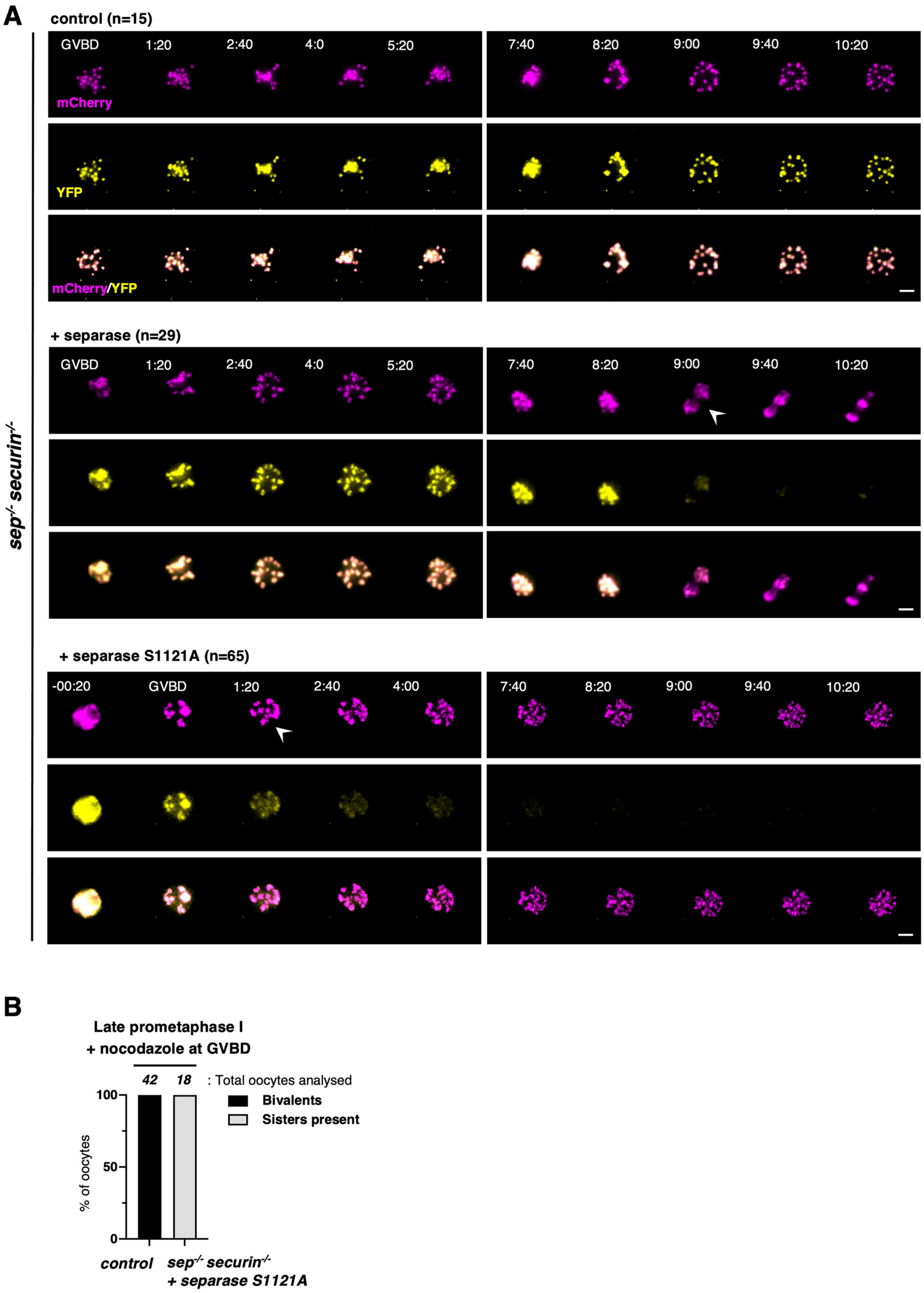
A
control (n=15)
GVBD 1:20 2:40 4:0 5:20 7:40 8:20 9:00 9:40 10:20
mCherry
YFP
mCherry/YFP
+ separase (n=29)
GVBD 1:20 2:40 4:0 5:20 7:40 8:20 9:00 9:40 10:20
sep-/- securin-/-
+ separase S1121A (n=65)
-00:20 GVBD 1:20 2:40 4:00 7:40 8:20 9:00 9:40 10:20
B
Late prometaphase I
+ nocodazole at GVBD
42 18 : Total oocytes analysed
Bivalents
Sisters present
% of oocytes
100
50
0
control
sep-/- securin-/-
+ separase S1121A

 © The Author(s)

**Figure EV3. Related to Fig. 3A,C; Movie EV2 and Appendix Fig. S1.**

Complete loss of separase control reveals absence of cohesin protection in early prometaphase I. (**A**) YFP and mCherry channels of selected time frames overlays shown in Fig. 3A. Time after GVBD is shown in hours:minutes (cleavage onset is indicated with an arrowhead). *n* is the number of oocytes analyzed. Scale bar (white) represents 20 µm. Complete movies are shown in Appendix Fig. S1. (**B**) Frequency of chromosome categories observed at late prometaphase I (6 h after GVBD) quantified from chromosome spreads of wild-type and *sep*$^{-/-}$*securin*$^{-/-}$ oocytes expressing separase S1121A shown in Fig. 3C. Oocytes were treated with nocodazole at GVBD. Total number of chromosomes quantified for each condition is indicated. sep: separase. Data information: Results shown were obtained from at least three independent biological replicates.

© The Author(s) 

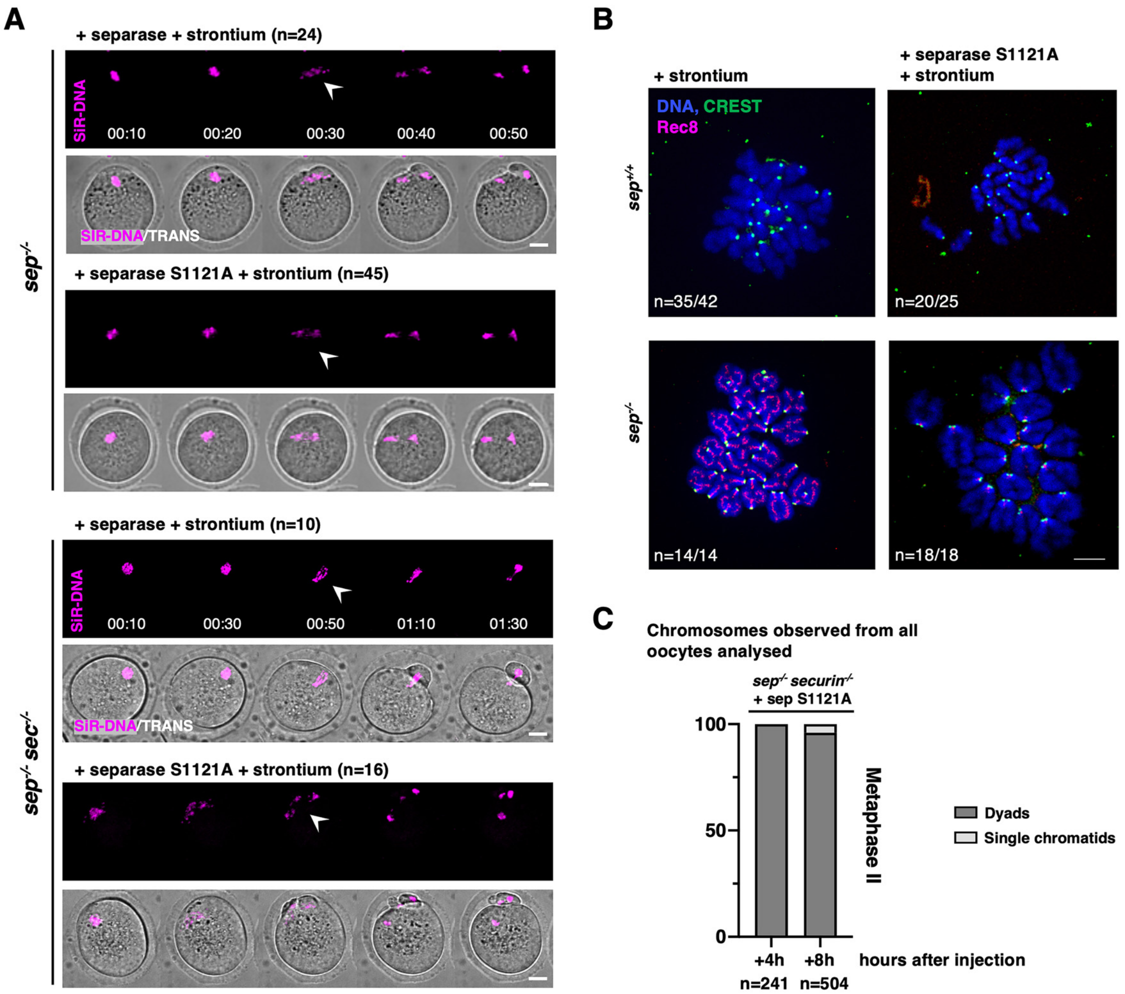


**Figure EV4. Related to Figs. 4B,D and 5B,D.**

When rescued in metaphase II, *sep*$^{-/-}$ and *sep*$^{-/-}$*securin*$^{-/-}$ oocytes can be activated to undergo meiosis II and separate chromosomes. (**A**) Live imaging movies of *sep*$^{+/+}$ (top) and *sep*$^{-/-}$ *securin*$^{-/-}$ (bottom) oocytes in Figs. 4B and 5B. Where indicated, oocytes were chemically induced with strontium to verify sufficient expression of wild-type separase or separase S1121A. For all conditions, oocytes were injected with the constructs around 16 h after GVBD, and were subjected to live imaging around 18 h after GVBD, corresponding to 5 min after oocytes were activated with strontium. Time after start of the movie is shown in hours:minutes (anaphase II onset is indicated with an arrowhead). n is the number of oocytes analyzed. Scale bar (white) represents 20 µm. (**B**) *sep*$^{+/+}$ (top) or *sep*$^{-/-}$ (bottom) oocytes were chemically induced with strontium and fixed for chromosome spreads around 20 h after GVBD (Metaphase II). Where indicated, oocytes were injected with the separase S1121A construct around 16 h after GVBD, and were fixed around 20 h after GVBD, corresponding to 45 min after oocytes were activated with strontium. Centromeres/kinetochores were stained with CREST (green), cohesin with anti-Rec8 antibody (red) and chromosomes with DAPI (blue). A representative spread and magnification of one chromosome (white dashed line squares, insert at the bottom right corner) are shown for each condition. n indicated the total number of chromosomes analyzed. Scale bars (white) represents 10 µm. (Related to Figs. 4D and 5D). (**C**) Frequency of chromosome categories observed at metaphase II quantified from *sep*$^{-/-}$*securin*$^{-/-}$ oocytes expressing separase S1121A. Oocytes were injected with the construct around 16 h after GVBD and were fixed for chromosome spreads at the indicated timings (4 h or 8 h after injections). Total number of chromosomes quantified for each condition, is indicated. sep: separase. (Related to Fig. 5D). Data information: Results shown were obtained from at least three independent biological replicates.

 © The Author(s)

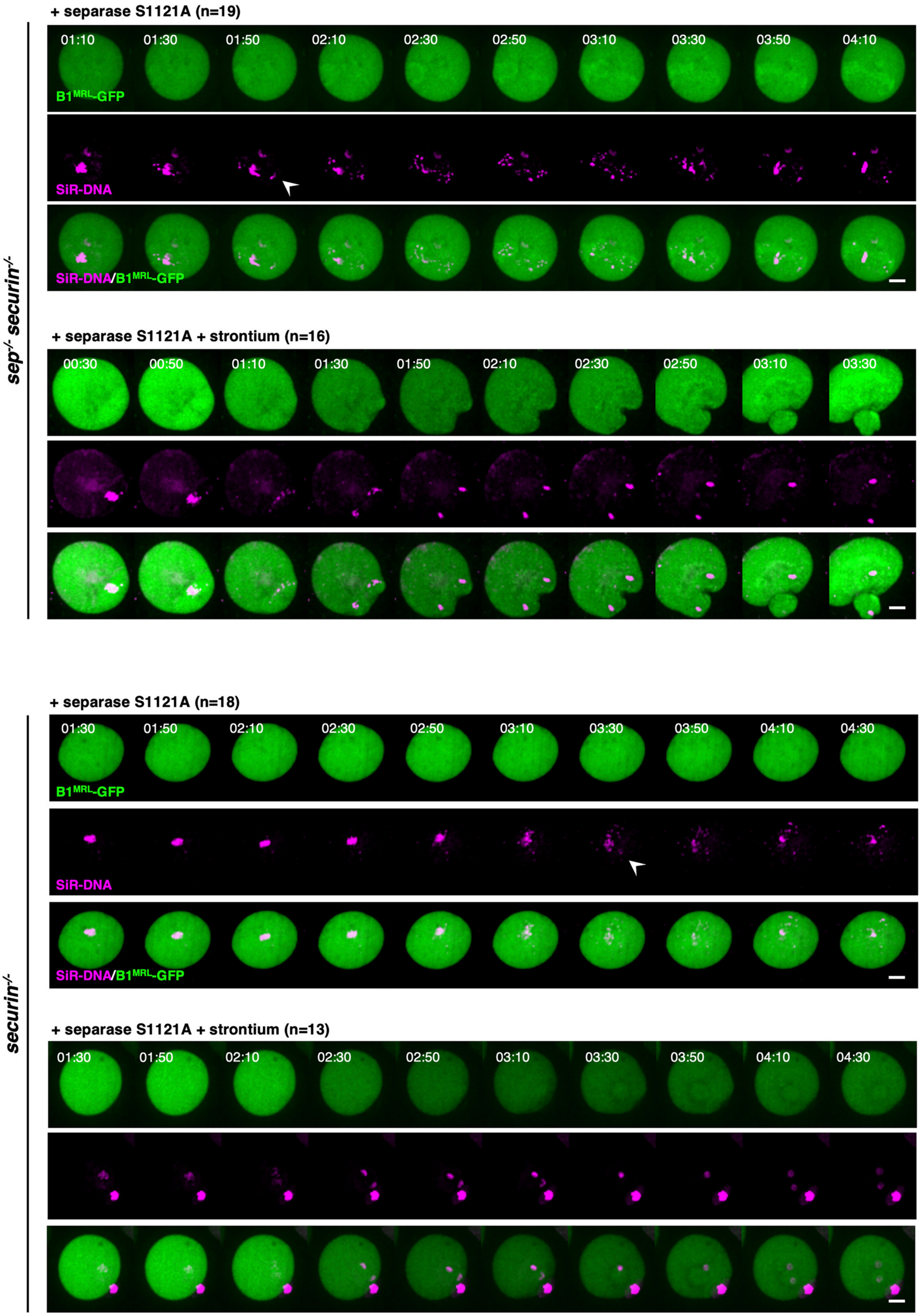
+ separase S1121A (n=19)
01:10 01:30 01:50 02:10 02:30 02:50 03:10 03:30 03:50 04:10
B1MRL-GFP
SiR-DNA
SiR-DNA/B1MRL-GFP
sep-/- securin-/-
+ separase S1121A + strontium (n=16)
00:30 00:50 01:10 01:30 01:50 02:10 02:30 02:50 03:10 03:30
+ separase S1121A (n=18)
01:30 01:50 02:10 02:30 02:50 03:10 03:30 03:50 04:10 04:30
B1MRL-GFP
SiR-DNA
SiR-DNA/B1MRL-GFP
securin-/-
+ separase S1121A + strontium (n=13)
01:30 01:50 02:10 02:30 02:50 03:10 03:30 03:50 04:10 04:30

◀ **Figure EV5. Related to Fig. 6D,F.**

Separase-out-of-control activation is independent of APC/C activation in meiosis II. Overlays of the GFP and Far-Red channels of the selected time frames shown in Fig. 6D,F. *sep*$^{-/-}$ *securin*$^{-/-}$ (top) and *securin*$^{-/-}$ (bottom) oocytes were injected with separase S1121A and the GFP-cyclin B1 MRL mutant around 16 h after GVBD, and were subjected to live imaging around 18 h after GVBD. Where indicated, oocytes were activated with strontium around 5 min before start of live imaging. Time after start of the movie is shown in hours:minutes (separation of chromosomes is indicated with an arrowhead). n is the number of oocytes analyzed. Scale bar (white) represents 20 µm. Data information: Results shown were obtained from at least three independent biological replicates.

 © The Author(s)
